# Advances of Brain Transcriptomics

**DOI:** 10.3390/genes13101831

**Published:** 2022-10-11

**Authors:** Olga E. Redina, Vladimir N. Babenko

**Affiliations:** Institute of Cytology and Genetics, Siberian Branch of Russian Academy of Sciences, 630090 Novosibirsk, Russia

Advancements in RNA sequencing technology in past decade have underlined its power for elucidating the brain gene networks responsible for various stressful factors, as well as the pathologies associated with both genetically determined neurodegenerative diseases and those acquired during the lifespan. As these exciting studies have continued to grow in recent years, we present a series of research papers reporting on progress and new findings based on the analysis of brain transcriptional activity. The brain transcriptome is the most evolved among tissues in terms of transcriptome plasticity and responses to various stimuli. In this Special Issue, we address a spectra of studies, including, but not limited to, animal models of social stress response and various brain-disease-related data/models, in which the identification of features in gene transcription profiles aids our understanding of the molecular mechanisms of socially significant disease development.

Publicly available databases allow researchers to test hypotheses and uncover additional developmental mechanisms of a variety of neuronal degenerative disorders that are actively studied, but remain poorly understood and undefeated. A group of bioinformaticians from Lomonosov Moscow State University, using RNA sequencing data freely available from the Sequence Read Archive, tested a hypothesis about the role of adenosine-to-inosine mRNA-editing patterns in the development of Parkinson’s disease (PD) [1]. They analyzed potential editing sites and showed decreased editing levels in the brains of patients with PD.

Autism spectrum disorder (ASD) is a neurodevelopmental pathology that impedes patients’ cognition, social skills, speech, and communication. In recent years, the prevalence of ASD has been steadily increasing; therefore, the identification of the molecular mechanisms underlying ASD occurrence and development is a socially important task. Since ASD is characterized by high heterogeneity and—accordingly—diverse etiology and clinical manifestations, the molecular mechanisms of its development have not yet been fully studied. Lee et al. [2] present a comprehensive study of the molecular basis of this pathology, carried out using integrated multilayer genomics data. The authors compared the RNA sequencing (RNA-seq) expression profiles of induced pluripotent stem cells (iPSCs), neural progenitor cells (NPCs), and neuron cells from samples from patients with and without ASD; their results suggest that ASD development occurs at the early stage of neural system development. The study allowed the authors to uncover variations in the regulatory cascades between samples from patients with and without ASD, as well as to determine a set of novel disease-associated genes and gene interactions; in particular, they highlighted the functional roles of *ELF3* and the interaction between *STAT1* and lncRNA *ELF3-AS 1* in ASD development.

Spinocerebellar ataxias (SCAs) are a heterogeneous group of dominantly inherited ataxias characterized by progressive cerebellar atrophy mainly due to the dysfunction in and loss of Purkinje cells. Szilvia E. Mezey and colleagues used a SCA14 mouse model to investigate Spinocerebellar ataxia type 14 (SCA14), which is a rare variant of SCAs caused by missense mutations or deletions in *PRKCG* gene encoding protein kinase C gamma (PKCgamma) [3]. Comparison of transcription profiles of the cerebella from control mice and PKCgamma-A24E mice—which were heterozygous and homozygous for a knock-in pseudo-substrate domain mutation in PKCgamma—allowed the authors to identify key genes and show involved metabolic pathways; these findings expanded our understanding of how mutated PKCgammas might be involved in SCA14 pathogenesis.

It is well known that human lifespans are full of different social conflicts, which can lead to the development of various neurological disorders. An animal model of chronic social confrontation shows that daily agonistic interactions lead male mice to form alternative patterns of social behavior depending on victories and defeats. This model of chronic social confrontation has proven to be an effective experimental approach in elucidating differences in the molecular mechanisms underlying the excitation of the brain neurons in chronically winning mice (winners) and chronically defeated mice (losers) compared with intact controls.

This Special Issue presents the results of a comparative analysis of gene expression profiles in the midbrain raphe nuclei (MRNs) of control male mice compared with winners and losers. It is known that MRNs contain a large number of serotonergic neurons associated with the regulation of numerous types of psycho-emotional states and physiological processes. The article by Redina and colleagues focuses on an analysis of the co-expression of DEGs and Tph2-encoding tryptophan hydroxylase 2, the rate-limiting enzyme of the serotonin synthesis pathway [4]. The results showed that Tph2 expression correlated with the expression of many DEGs, associated with behavior, learning, memory, and synaptic signaling. Many common DEGs correlated with Tph2 expression in the MRNs of both aggressive and defeated mice were downregulated. Based on the results obtained, it has been suggested for the first time that CRH and TRH (locally produced in MRNs)—which control the synthesis of corticotrophin- and thyrotropin-releasing hormones, respectively—may be involved in the serotonergic regulation of brain processes during chronic social conflict.

Another article in this Special Issue discusses the contribution of the altered expression profiles of genes encoding the solute carrier (SLC) transporters, which may serve as markers of altered brain metabolic processes and neurotransmitter activities in psychoneurological disorders [5]. The article compares the expression profiles of the Slc genes in the ventral tegmental area (VTA), the nucleus accumbens (NAcc), and the prefrontal cortex (PFC) of both control and aggressive male mice with psychosis-like behavior, induced by repeated experience of aggression accompanied with wins in daily agonistic interactions. The results demonstrated that altered Slc gene activity may lead to a restructuring of metabolic and neurotransmitter processes in a way which is specific for each brain region in the male mice with repeated experience of aggression.

As a continuation of these studies, we include a report by the same group of authors on changes in the level of gene expression in the dorsal striatum in aggressive (winners) and aggression-deprived (AD) male mice [6]. Earlier work by the authors showed that AD mice displayed a higher aggressive behavior score compared with the aggressive group. The results presented in this Special Issue show for the first time that a number of gene-encoding transcription factors did not restore transcription levels within 14 days of aggression deprivation. The authors outlined specific TF-activator gene networks associated with transcriptional repression in affected species compared with controls and concluded that aggressive phenotype selection with a positive reward effect (winning) manifested in an addiction model, which featured a distinct opioid-related withdrawal effect in the AD group.

A study on the impact of social stress on brain plasticity is presented by a team of authors from the University of Illinois at Urbana-Champaign [7]. Using a porcine maternal immune activation (MIA) model, it was shown that activation of maternal immunity during pregnancy and social stress during weaning can alter brain plasticity in the offspring’s hippocampus. An analysis of differences in the expression profiles of hippocampus genes in groups of control and experimental animals allowed the authors to conclude that the terpenoid backbone biosynthesis and cocaine addiction pathways played important roles in a three-way interaction between sex, maternal immune activation, and weaning stress.

This Special Issue presents the results of another experiment by the same group of authors, which aimed to study the impact of proinflammatory challenges caused by maternal immune activation (MIA) and postnatal exposure to drugs of abuse (morphine) on the prefrontal cortex molecular pathways [8]. The range of interacting effects on the prefrontal cortex pathways were uncovered, providing new insights into the interplay between the effects of MIA and morphine.

Another article in this Special Issue is devoted to the study of transcription profiles in the brain of Takifugu rubripes fish under hypoxia and normoxia to identify pathways involved in regulating brain metabolism under chronic hypoxic stress. The results of the work also provided new insights into the adaptive molecular mechanisms that arise when the brain responds to hypoxic stress [9].

Long noncoding RNAs (lncRNAs) play an important role in the control of many physiological and pathophysiological processes. Differential hypothalamic expression of several lncRNAs was found to be associated with hypertension and the behavior/neurological phenotypes of ISIAH rats; this was characterized as a rat model for a stress-sensitive form of hypertension [10].

In summary, the current Special Issue “Advances of Brain Transcriptomics” uncovers the molecular mechanisms related to transcriptional changes that accompany the development of neurodegenerative diseases or those involved in response to various social or environmental stressors. This knowledge paves the way for the next steps of further research in this field, which will expand the boundaries of our understanding of the processes occurring in the brain under conditions of pathological changes or under the influence of adverse factors.

## Data Availability

Not applicable.
